# Identification of a VHL gene mutation in atypical Von Hippel-Lindau syndrome: genotype–phenotype correlation and gene therapy perspective

**DOI:** 10.1186/s12935-021-02386-w

**Published:** 2021-12-19

**Authors:** Dali Tong, Yao Zhang, Jun Jiang, Gang Bi

**Affiliations:** Department of Urology, Daping Hospital, Army Medical University, Chongqing, 400042 People’s Republic of China

**Keywords:** Von Hippel Lindau (VHL), Gene mutation, Carcinoid, Neuroendocrine tumor

## Abstract

**Background:**

Classical von Hippel Lindau (VHL) disease/syndrome includes CNS hemangioblastoma, renal or pancreatic cysts, pheochromocytoma, renal carcinoma and exodermic cystadenoma. The syndrome is caused by mutation of VHL tumor suppressor gene. The most prevalent mutations are present in VHL syndrome. To date, > 500 mutations of gene related to the progression of VHL syndrome have been reported. VHL gene mutation presented in single lung or pancreatic tumor has been reported occasionally, but there is no report of both.

**Methods:**

In this paper, we used CT scan, pathological and genetic examination methods to diagnose a rare atypical VHL syndrome.

**Results:**

We reported a rare case of atypical VHL syndrome with authenticated VHL mutation at p.Arg167Gln, that was associated with not only bilateral pheochromocytoma but also lung carcinoid and neuroendocrine tumor of pancreas. Based on literature reviews, the patient was recommended to be further subjected to octreotide-based radionuclide therapy.

**Conclusions:**

Combined with gene detection and clinical diagnosis, we found the inherent relationship between VHL genotype and phenotype, and constructed the standard diagnosis and treatment process of disease with rare VHL mutation from the perspective of gene therapy.

**Supplementary Information:**

The online version contains supplementary material available at 10.1186/s12935-021-02386-w.

## Introduction

Von Hippel Lindau (VHL) gene locates in chromosome 3p25-p26 and encodes VHL proteins, among which pVHL19 and pVHL30 are tumor-suppressing proteins and defined as pVHL. VHL protein contains α-domain and the β-domain, of which the α-domain plays key role in maintaining VHL protein stability by binding to translational elongation factor C, and the β-domain acts as substrate recognizer for pVHL. The β domain binds with hypoxia-inducible factor (HIF) subunit and controls HIF subunit degradation. VHL mediates tumor invasion and metastasis by regulating HIFs protein expression [1, 2].

Up to now, according to data from the Human Gene Mutation Database, > 500 VHL gene mutations leading to VHL syndrome have been reported [3, 4]. The majority of VHL mutations are deletion and missense, of which the most prevalent mutations include R167/161, V155, Y98, G114, F76, P86/81, L158 and C162. These mutation sites locate in the HIF-1α- and elongation factor C-binding domains [5–7]. pVHL binding to HIF-1α may lead to the degradation of HIF protein and reverse oncogenesis. Previous studies have demonstrated that pVHL-S111 and pVHL-H115 act as important regulators causing HIF degradation.

The VHL syndrome is caused by germline mutations of VHL gene and featured by group of multiple tumors, related to a high morbidity and mortality and exhibition of diverse phenotypes. Classical von Hippel Lindau (VHL) disease/syndrome includes CNS hemangioblastoma, renal or pancreatic cysts, bilateral pheochromocytoma, renal carcinoma and exodermic cystadenoma. Since the diversity of clinical symptoms of VHL disease, the diagnosis and follow-up of VHL syndrome remain a challenge and need multidisciplinary treatment approach. Updated experience is the detection of tumors including pheochromocytomas and islet cell tumors in patients, based on symptoms including hypertension, hypoglycemia, cardiac arrhythmias, and carcinoid syndrome [8–11].

The greatest tumor diameter associated with *VHL* genotype was recently reported. During median follow-up of 4.5 years, patients with missense *VHL* gene mutations are more prone to developing metastatic disease. However, missense *VHL* gene mutation eventually requiring surgical intervention are not very common in clinical practice. In fact, patients with non-missense mutations are rarely required for surgical intervention due to low-risk disease progression [12].

Carcinoids, as a kind of rare tumors, are originated from enterochromaffin cells, which can be found wide distribution in the whole body. The incidence in every year is two cases per 100 thousand people, accompanied with the gastrointestinal (60%) and bronchopulmonary tracts (25%) as the most common primary sites. Carcinoid of the gallbladder or the biliary tree have been found in VHL disease [13, 14]. However, there is no direct evidence suggesting a link between VHL gene mutations and carcinoids. Here, we reported a case of multiple-site carcinoids in the lung and pancreas, and provided genetic evidence of potential association between the carcinoid and VHL gene mutation [15].

In this paper, we found that authenticated VHL mutation p.Arg167Gln is not only associated with bilateral pheochromocytoma but lung carcinoid and neuroendocrine tumor of pancreas, which have not been reported.

## Methods

### Ethical review and patient consent

The study was approved by the Research Ethics Committee of Daping Hospital, Army Medical University (Chongqing, China) and obtained the written informed consent from the patient for the use of medical records associated with related images. The clinical samples and images were provided by the Department of Pathology and Medical Imaging. All methods were performed in accordance with the relevant guidelines and regulations. Legal guardian of the patient has signed informed consent for publication of identifying information in an online open-access publication.

## Clinical data

### Case presentation

#### First onset

A 19-years-old female was first found with occupation about 6.2 cm × 5.7 cm in the right adrenal five years ago based on CT scan at a regular physical examination, accompanied with fever and headache with high blood pressure. Tumor resection was performed, and pathological diagnosis was right pheochromocytoma. The patient was regularly subjected to follow-up.

#### Secondary onset

During this hospitalization, CT scan found multiple tumor occupation in left adrenal and paraganglion region zones with round nodular shadows. Meanwhile, she had fever and headache with high blood pressure. Based on medical history and examination, she was diagnosed as left pheochromocytoma. Meanwhile, the occupying lesions in right lung and pancreas were found, located in extrabasal segment of inferior lobar and head respectively.

#### Case characteristics

The case characteristics were summarized: 1. A 12y female; 2. Tumor occupation was first found in right adrenal; 3. Tumor resection was performed and the pathological diagnosis was pheochromocytoma; 4. Multiple round nodular shadows were found in left adrenal and paraganglion region zones after five years; 6. The symptoms were fever and headache with high blood pressure; 7. The diagnosis was pheochromocytoma (PCC) and paraganglioma (PGL) (Combined PPGLs); 8. The occupying lesions located in right lung and pancreas were scanned and diagnosed with masses.

### Diagnostic method

The patient was followed up regularly. Based on personal medical history, symptoms and imaging examinations, the patient was diagnosed as PPGLs combined with right lung and pancreas occupation.

**Fig. 1:**
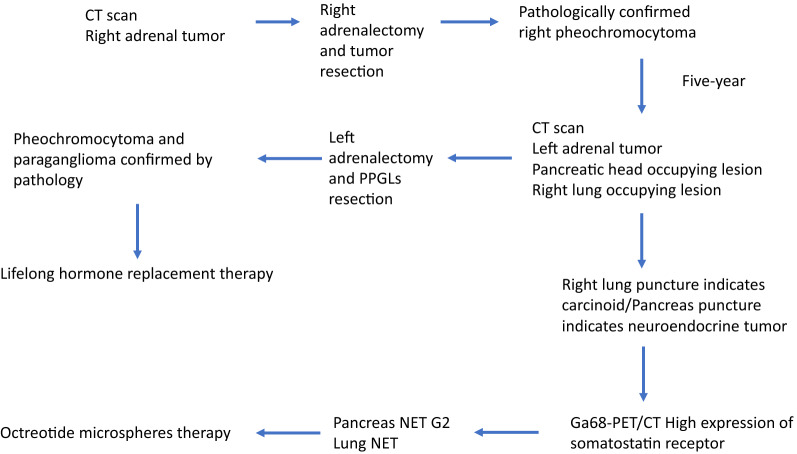
Schematic program of the medical history, diagnosis and treatment workflow of patient in whole process. Specially, the patient underwent two operations aiming to pheochromocytoma and paraganglioma (PPGLs), followed by findings of lung and pancreas neuroendocrine tumor NET and subjected to octreotide-based radionuclide therapy

Multidisciplinary team discussion based on professional board indicated the following views: (1) The diagnosis was possibly considered as atypical VHL type II. Further qualitative diagnosis relied on genetic examination; (2) Other lesions like retina tumor should be examined. (3) PPGLs was surgically resected for pursuing pathological diagnosis. (4) The occupying lesions in right lung and pancreas were performed with puncture biopsy for further diagnosis. The diagnosis and treatment workflow were shown in Fig. 1.

#### CT scan

64-slice spiral CT was used to conduct examination of potential tumors. After receiving basic respiratory training, patients hold their breath to reduce respiratory artifacts. Conventional plain CT scanning of whole abdominal was performed to localize masses with parameters 120 kVp, 200 mA. After intravenously injected by non-ionic iodinated contrast medium, the patient received enhanced scanning. The contrast-enhanced CT imaging was performed following the manufacturer's instructions. The tumor was preliminarily positioned and qualitatively determined. The reconstruction slice thickness was dependent on the tumor size. In the image analysis, tumors were identified by two imaging physicians with experience. Diagnostic criteria: positioning and qualitative judgment of tumors. CT diagnosis of cancer requires at least two doctors with the corresponding titles of radiology to evaluate the CT image, including the location, shape, size, cystic solidity, blood supply, degree of enhancement and peritoneal implantation [16].

#### Pathological diagnosis

All the tissue specimens were obtained following by standard surgical oncology procedure and performed according to standard hematoxylin and eosin (H&E) staining procedures and cytology process. Pathological diagnosis is based on two professional licensed pathologists.

#### Gene diagnosis

DNA was extracted from the peripheral blood of the patient. 1 mL of peripheral blood was collected to extract genomic DNA (Qiagen, Germantown, MD, USA). The entire exomes were captured and sequenced on an Illumina (San Diego, USA) platform. Paired end reads were aligned to the human genome (build GRCh37/hg19), and variants were called, annotated, and filtered using a custom-made pipeline. Segregation analysis was performed to establish a diagnosis. Total DNA was used for screening the potential mutations in the following genes: SDHAF2 (succinate dehydrogenase complex assembly factor 2), SDHB (succinate dehydrogenase subunit B), SDHC (succinate dehydrogenase subunit C), SDHD (succinate dehydrogenase subunit D), MAX (MYC associated factor X), NF1 (neurofibromin 1), RET, and VHL (Von Hippel–Lindau) using Target Capture-Based Deep Sequencing (BGI Health, Shenzhen, Guangdong, China). The genes sequence was amplified by polymerase chain reaction (PCR), and sequencing was performed from both the DNA strands of the entire coding region and the intron and junction regions. The product of the sequencing was compared to a reference sequence of GRCh37 human genome [17, 18].

## Results

### The results of diagnosis/detection

Genetic surveillance assays were performed based on blood sample. Gene mutation and tumor mutation burden analyses indicated germline pathogenic mutation of VHL gene (c.500G > A, p.Arg167Gln) (Table 1). Furthermore, Genetic surveillance assays were performed based on tissue sample. Gene mutation and tumor mutation burden analyses indicated somatic pathogenic mutation of VHL gene (c.500G > A, p.Arg167Gln). Additionally, we also made the raw data of the VHL mutations characterized by gene expression available. We screened the germline and somatic VHL mutations at p.Arg167Gln associated with other somatic mutations including included COL4A6 (c.128C > T, p.Pro43Leu), HMGCL (c.647A > G, p.Asp216Gly), KIFC1 (c.935G > A, p.Arg312His), KPNA7(c.659C > T, p.Thr220Met), KSR2(c.2720G > A, p.Arg907His), MSX2(c.387G > T, p.Met129Ile), PARP4(c.3509C > T, p.Thr1170Ile), RER1(c.209C > T, p.Ala70Val), SCN10A(c.5454G > C, p.Leu1818Phe) based on genetic examination. The raw results were put into the Additional files 1, 2, 3, 4, 5, 6, 7, 8, 9, 10.

**Table 1:** Genetic surveillance assays were performed based on blood sample

Gene	Mutation type	Nucleotide change	Amino acid change	Amino acid change	Rate(%)	Chr	Exon	Initial position	Terminal position	Transcript number
VHL	Missense	c.500G > A	p.Arg167Gln	p.R167Q	50.5	3	3|3	10,191,507	10,191,507	NM_000551.3

Gene mutation and tumor mutation burden analysis indicated germline pathogenic mutation of VHL gene (c.500G > A, p.Arg167Gln)

Also, tumor mutation burden (TMB) is defined as the total number of somatic gene coding errors, base substitution, gene insertion or deletion errors detected per million bases, based on removing germline mutations in tumor genome. We examined 0.18/Mb in somatic mutation in whole tumor genome. According to Tables1 and 2, we found that the mutation rate of VHL p.Arg167Gln reached 50.5% in germline mutation and is much higher than other mutations in somatic mutation. Meanwhile, the mutation of VHL p.Arg167Gln is the only pathogenic mutation leading to VHL syndrome. There is no other mutations in the VHL gene except p.Arg167Gln in the cases. The other major gene mutations included COL4A6 (c.128C > T, p.Pro43Leu), HMGCL(c.647A > G,p.Asp216Gly),KIFC1(c.935G > A,p.Arg312His), KPNA7(c.659C > T,p.Thr220Met), KSR2(c.2720G > A,p.Arg907His), MSX2(c.387G > T, p.Met129Ile), PARP4(c.3509C > T, p.Thr1170Ile), RER1(c.209C > T, p.Ala70Val), SCN10A(c.5454G > C,p.Leu1818Phe). Except SCN10A, other genes have been verified to be related to carcinogenesis or cancer progression [19–26]. There are no further reports about the relationship between these detected mutations and cancer except PARP4(c.3509C > T, p.Thr1170Ile). Previous study found that rare variants containing T1170I in the PARP4 gene were detected at significant high frequency in participants with primary thyroid and breast cancers while their frequencies were only 0.5% in controls. PARP4 may function as a tumor suppression and be identified as a possible susceptibility gene of primary thyroid and breast cancer [25]. However, up to now, there is no direct evidence about the relationship of these major gene mutations with VHL syndrome (Table2).

**Table 2:** Genetic surveillance assays were performed based on tissue sample

Gene	Mutation type	Nucleotide change	Amino acid change	Amino acid change	Rate(%)	Chr	Exon	Initial position	Terminal position	Transcript number
COL4A6	Missense	c.128C > T	p.Pro43Leu	p.P43L	10.5	X	3|45	107,553,994	107,553,994	NM_033641.2
HMGCL	Missense	c.647A > G	p.Asp216Gly	p.D216G	29.6	1	7|9	24,134,728	24,134,728	NM_000191.2
KIFC1	Missense	c.935G > A	p.Arg312His	p.R312H	24.6	6	7|11	33,372,807	33,372,807	NM_002263.3
KPNA7	Missense	c.659C > T	p.Thr220Met	p.T220M	5.2	7	6|10	98,786,164	98,786,164	NM_001145715.2
KSR2	Missense	c.2720G > A	p.Arg907His	p.R907H	8.6	12	19|20	117,907,506	117,907,506	NM_173598.4
MSX2	Missense	c.387G > T	p.Met129Ile	p.M129I	9.9	5	2|2	174,156,169	174,156,169	NM_002449.4
PARP4	Missense	c.3509C > T	p.Thr1170Ile	p.T1170I	10.4	13	29|34	25,016,762	25,016,762	NM_006437.3
RER1	Missense	c.209C > T	p.Ala70Val	p.A70V	5.0	1	4|7	2,330,876	2,330,876	NM_007033.4
SCN10A	Missense	c.5454G > C	p.Leu1818Phe	p.L1818F	6.8	3	27|27	38,739,257	38,739,257	NM_006514.3
VHL	Missense	c.500G > A	p.Arg167Gln	p.R167Q	48.0	3	3|3	10,191,507	10,191,507	NM_000551.3

Gene mutation and tumor mutation burden analysis indicated somatic pathogenic mutation of VHL gene (c.500G > A, p.Arg167Gln). Tumor mutation burden was 0.18/Mb. There is no other mutations in the VHL gene except p.Arg167Gln in the case. The other major gene mutations included COL4A6 (c.128C > T, p.Pro43Leu), HMGCL (c.647A > G, p.Asp216Gly), KIFC1 (c.935G > A, p.Arg312His), KPNA7(c.659C > T, p.Thr220Met), KSR2(c.2720G > A, p.Arg907His), MSX2(c.387G > T, p.Met129Ile), PARP4(c.3509C > T, p.Thr1170Ile), RER1(c.209C > T, p.Ala70Val), SCN10A(c.5454G > C, p.Leu1818Phe)

**Fig. 2:**
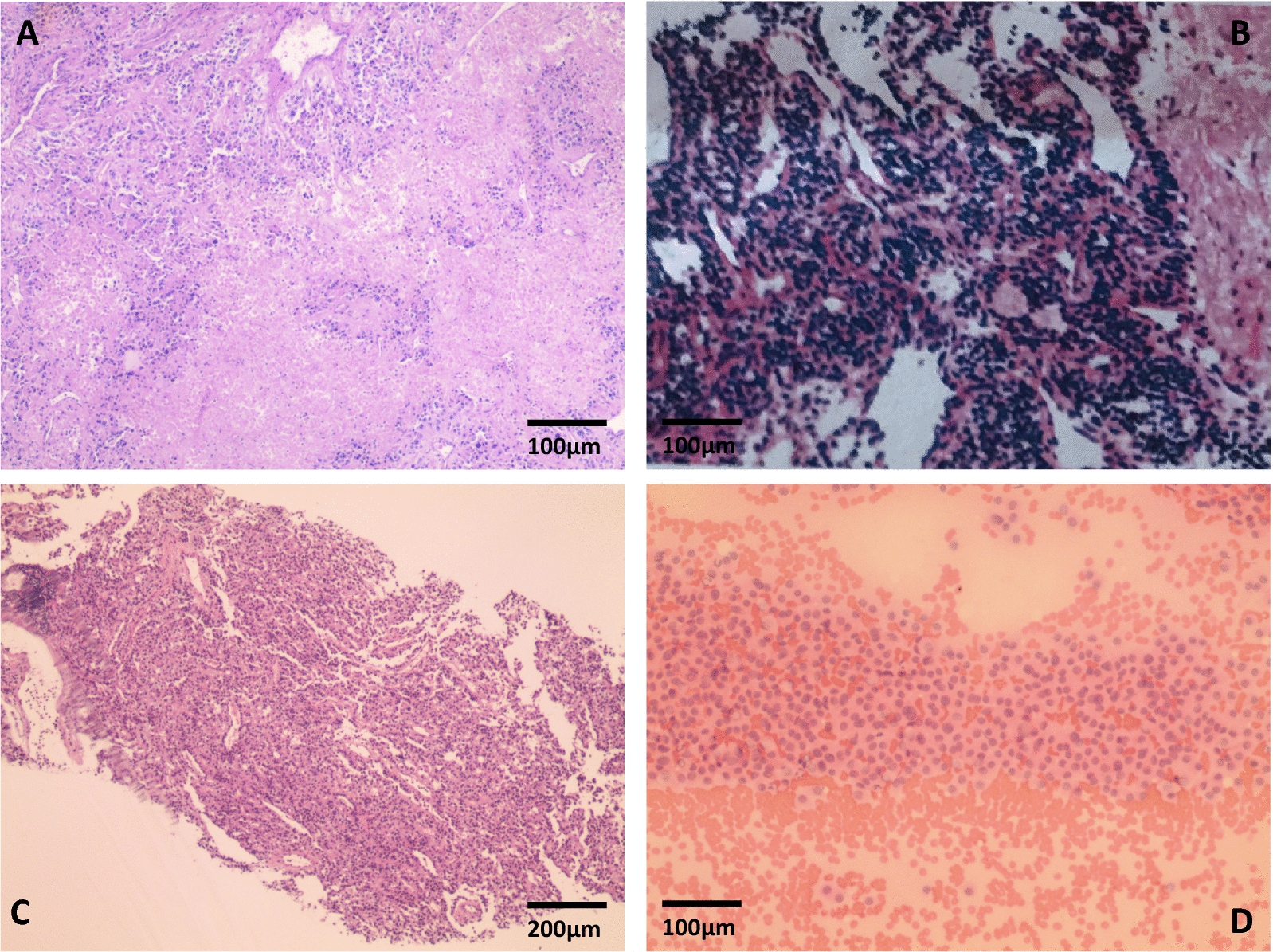
The tissues of left adrenal (**A**), pancreas (**B**) and lung (**C**) masses after surgical resection or puncture are subjected to hematoxylin and eosin (HE) staining. Pathological diagnoses are PPGL (**A** Magnifications: X100), pancreas neuroendocrine tumor (NET) G2 (**B** Magnifications: X100), lung neuroendocrine tumor (NET) carcinoid (**C** Magnifications: X40) respectively. Lung cytology examination indicated lung neuroendocrine tumor (NET) carcinoid (**D** Magnifications: X100)

The tissues of left adrenal (Fig. 2A), pancreas (Fig. 2B) and lung (Fig. 2C) masses obtained from surgical resection or puncture are subjected to hematoxylin and eosin (HE) staining. Pathological diagnoses are PPGLs, pancreas neuroendocrine tumor (NET) G2, lung neuroendocrine tumor (NET) carcinoid respectively. Lung cytology examination indicated lung neuroendocrine tumor (NET) carcinoid (Fig. 2D).

**Fig. 3:**
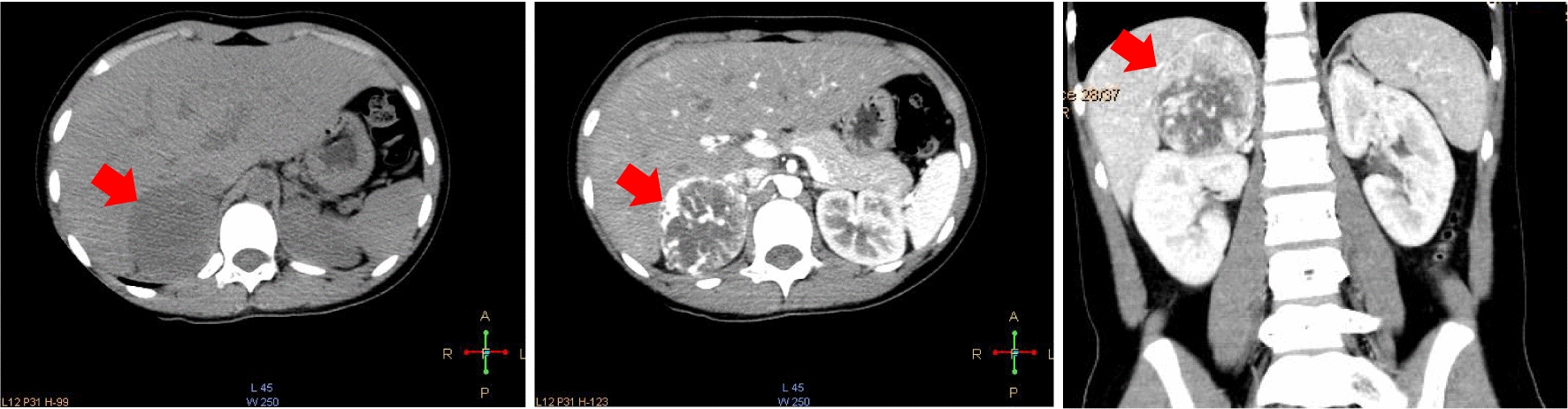
Transverse section and coronal section of CT scans indicated right pheochromocytoma, which were labelled as red arrow. CT scans indicated that tumor enhancement and central necrosis were obvious

Transverse section and coronal section of CT scans indicated right mass. The CT scans showed primary tumor around 6 cm in right adrenal zone. CT scans indicated that tumor enhancement and central necrosis were obvious. According to radiological analysis, pheochromocytoma was preferentially considered (as indicated by red arrows, Fig. 3).

**Fig. 4:**
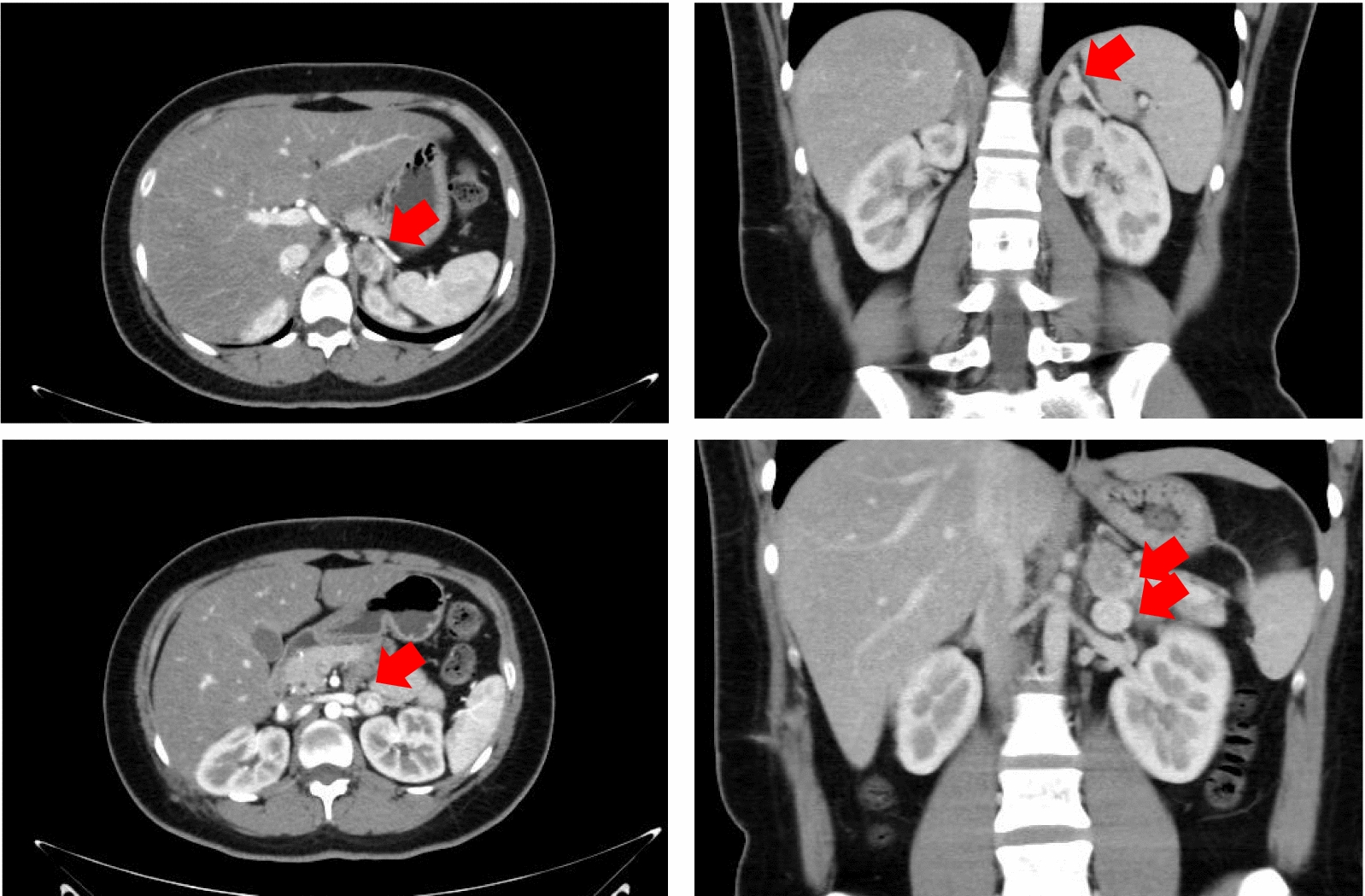
Transverse section and coronal section of CT scans indicated multiple pheochromocytoma and paraganglioma (PPGL) located in left side, which were labelled as red arrow. CT scans indicated that tumor enhancement and central necrosis were obvious

Three tumors were detected again in the left side around vessel after 5 years. Transverse section and coronal section of CT scans indicated multiple PPGLs located in left side, which were labelled as red arrows. CT scans indicated that tumor enhancement and central necrosis were obvious (Fig. 4).

**Fig. 5:**
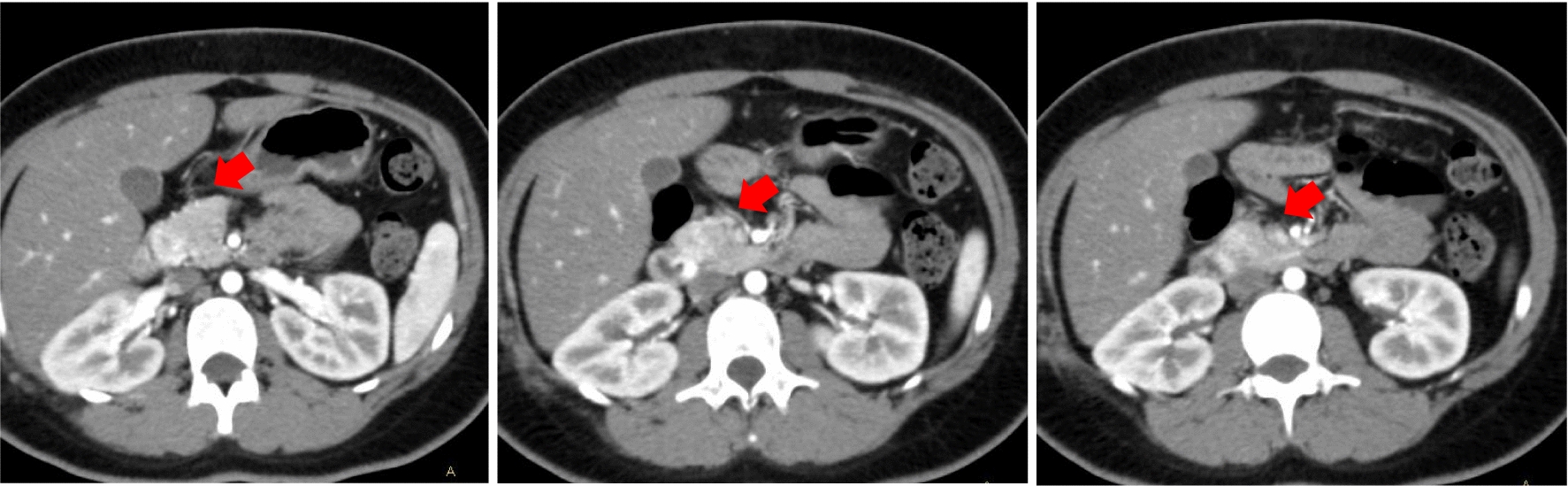
Transverse section CT scans indicated occupied lesion located in head of pancreas, which were labelled as red arrow. CT scans indicated that the tumor boundary was unclear and closely related to the surrounding organs. The enhancement was obvious. The pathological diagnosis was neuroendocrine tumor (NET) G2

**Fig. 6:**
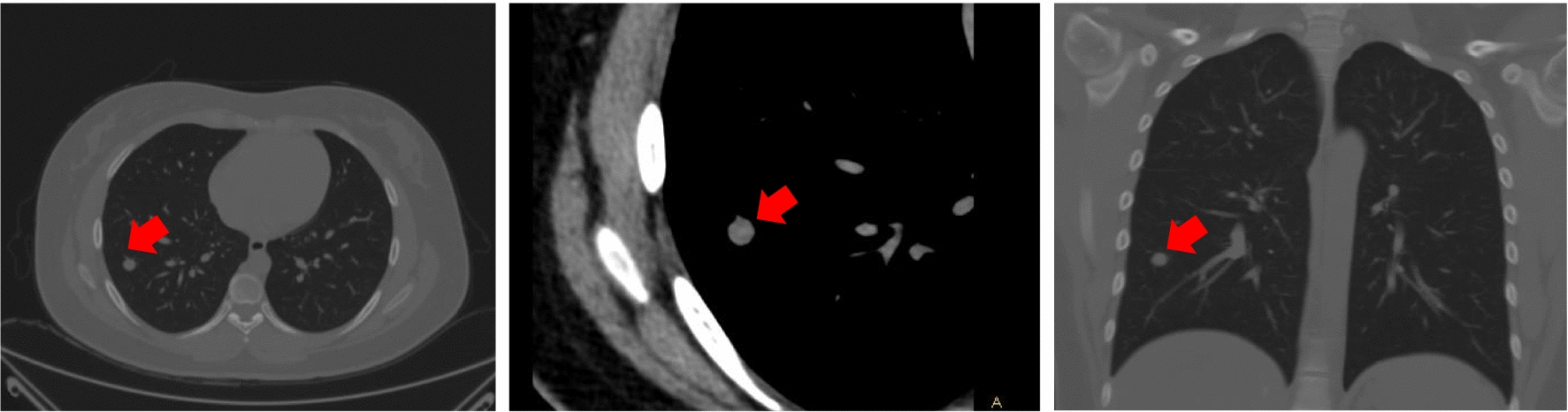
Transverse section and coronal section of CT scans indicated occupied lesion located in right lung, which were labelled as red arrow. The pathological diagnosis was neuroendocrine tumor (NET) carcinoid

Meanwhile, transverse section CT scans indicated occupied lesion located in head of pancreas, which were labelled as red arrow. CT scans indicated that the tumor boundary was unclear and closely related to the surrounding organs. The enhancement was obvious. The pathological diagnosis was neuroendocrine tumor (NET) G2 (Fig. 5). Moreover, Transverse section and coronal section of CT scans indicated occupied lesion located in right lung, which were labelled as red arrow. The pathological diagnosis was neuroendocrine tumor (NET) carcinoid (Fig. 6).

### The results of treatment

Before the operation, the patient had not received any drug treatment including targeted therapy or immunotherapy. Surgical resection was recommended to relieve the symptoms. After resection bilateral PPGLs, the patient received lifelong hormone replacement therapy and octreotide-based radionuclide therapy. We pursued literature reviews on treatment approaches aiming to unresectable NET, are very few and limited. The immunohistochemical staining indicated pancreas NET with somatostatin receptor2 (SSTR2) (Positive above 95%) and Ki67(+ 13%) expression (Fig. 2).

Up to now, several potential therapies were recommended to treat pancreas NET: (1) Everolimus and sunitinib were used as small molecule-targeted drugs for long-term treatment, aiming to prolong progression free survival of tumor; (2) Long-acting somatostatin anologues (SSAs) like octreotide and lanruide served to treat low Ki67 (below 10%) and positive SSTR2; (3) Chemotherapy based on STZ + 5-FU/TMZ/EP plans aimed to large tumor burden and fast growth with low-grade NET and low-differentiation neuroendocrine carcinoma (NEC); (4) The full name of PRRT is peptide receptor-mediated radionuclide therapy, which is a specific isotope therapy for neuroendocrine tumors. It aims to somatostatin receptor expressed in neuroendocrine tumor cells. The radioisotope can kill tumor cells by labeling the radioisotope with somatostatin analogue and injecting it into the patient's body. At present, PRRT is mainly applied to well differentiated primary or secondary NETs [27–29]. Peptide receptor radionuclide therapy (PRRT) with 177Lu-DOTATATE was recently approved by the FDA for the treatment of gastroenteropancreatic NET. Moreover, lung NEN includes well differentiated neuroendocrine tumors (NET) classified as typical carcinoids or atypical carcinoids. Experienced multidisciplinary NET teams may consider PRRT alongside everolimus as an option for patients with advanced somatostatin receptor-positive lung typical/atypical carcinoids [30, 31]. The patient experienced surgical PPGLs section and remained pancreas NET G2 and lung carcinoid with positive SSTR2 and slow growth. Due to low toxicity, PRRT has been verified to achieve worthwhile clinical and biochemical responses and encourage survival for well differentiated NETs. Furthermore, compared to I131-MIBG therapy, PRRT suggests radiation-safety and -efficacy advantage in NETs and PPGLs [32]. The patient was recommended to be subjected to octreotide-based radionuclide therapy, and long-term efficacy needed further evaluation.

## Discussion

VHL syndrome presents diverse phenotypes in some patients with either RCC or PCC, while some with both. Generally, VHL syndrome can be classified into two groups: Group 1 (without PCC) and Group 2 (with PCC). Group 2 disease can be further divided into three subtypes: 1. Type 2A: Patients with PCC and HB in the CNS, but without RCC; 2. Type 2B: patients with Pheo, RCC and other CNS tumors, 3. Type 2C: Patients with PCC alone [33, 34]. PCC or PGL induced by VHL mutation appears at different ages with a median age of 30 years. PCC always have bilateral, multiple extra-adrenal and asymptomatic features.

VHL disease is always associated with NET. In pancreatic abnormalities, most patients exhibit different types of pancreatic lesion including pseudocyst, serous cystadenomas and NETs [35]. Clear cell neuroendocrine tumor is a rare in non-VHL patients and most often observed in patients with VHL disease. Clinicopathological detection indicated clear cell NET G1 of the gallbladder occurs in VHL-mutated patients. Moreover, α-inhibin was detected in VHL case associated with clear cell NET tumor, suggesting that α-inhibin may be a biomarker and an promoter between clear cell NET and VHL [13].

Based on FISH data, allelic deletions of VHL gene not only exhibit in the VHL-related HB and PCC, but in the primary and metastatic carcinoids. Although carcinoids have similar histological and cellular components to VHL-associated NET, it is not well known about relationships between VHL gene mutations and carcinoids development. For example, the heterozygous allelic deletions of VHL gene were demonstrated in the primary lung and metastatic brain carcinoids, which provide genetic evidence for potential role of VHL mutation in the pathogenesis of carcinoids [15]. According to the two-hit hypothesis leading to tumorigenesis, recent studies have demonstrated that VHL-mutated tumors always exhibit loss of heterozygosity. In the process, an allele presents mutation with a manner of inherited germline and further merges with deletion of the wild-type allele. VHL p.Arg167Gln mutation is a likely contributory cause for VHL group 2 rather than group 1. The mutation approaches to cause PCC and HB located in nervous system such as the retina, cerebellum and spinal cord. Moreover, PCC located in (c.500G > A; p.Arg167Gln) might be either functional or non-functional. Patients carrying p.Arg167Gln mutation may present with bilateral pheochromocytomas but without episodic headache, sweating, and tachycardia. The laboratory examination result indicates higher levels of urine normetanephrine compared to metanephrine levels [7, 36]. We found a case with authenticated VHL mutation p.Arg167Gln is not only associated with bilateral PPGLs but lung carcinoid and neuroendocrine tumor of pancreas, which have not been previously reported. These clinical manifestations are also different from classical multiple endocrine neoplasia type I, II III and IV.

In order to better illustrate and generalize the significance of VHL mutations in VHL syndrome or genetic pheochromocytoma and paraganglioma (PPGLs) worldwide, we screened recent literatures about VHL mutations in VHL syndrome or genetic PPGLs. According to review from Joakim Crona, et al. [37], the proportion of patients with hereditary PPGL was estimated to be as high as 40%, reflecting a steady increase in the number of susceptibility loci. Between 1 and 13% of PPGLs have germline VHL mutations [38–42], whereas the cumulative frequency of RET, NF, TMEM, and MAX is reported to between 1 and 11%. About 12% to 16% of PPGLs are expected to have SDHx or FH mutations, including mainly PGLs (22% to70%). Carriers of VHL mutations have a reduced life expectancy of 60 to 65 years. However, PPGLs were classified as the cause of death in only 2% of patients with von Hippel–Lindau (VHL) syndrome (1/67) [43].

In recent reviews, Michael Reich et al. concluded the data from single-centre cohort study of Germany including 216 patients with clinically expected VHL disease due to positive family history or the presence of VHL typical tumors [44]. In total, 42 different rare VHL gene variants were detected, including truncating (Deletion, VHL gene deletion, Deletion Exon 1 and 2, Deletion Exon 2 and 3, Deletion Exon 1, Deletion Exon 2, Deletion Exon 3), Splice (c.464-2A > G), Frameshift (c.220del, c.408del, c.493del), Nonsense (c.394C > T, c.481C > T, c.490C > T, c.548C > A, c.555C > A), In frame (c.227_229del), Missense (c.233A > G, c.235C > G, c.238A > C, c.239G > T, c.254T > C, c.256C > G, c.257C > A, c.262T > A, c.266 T > C, c.269A > T, c.292 T > C, c.319C > G, c.320G > A, c.335A > G, c.386T > C, c.388G > A, c.395A > C, c.407T > C, c.461C > T, c.463G > C, c.475A > G, c.486C > G, c.491A > T, c.499C > T, c.562C > G), Synonymous (c.93G > A). Marie Louise Mølgaard Binderup concluded the data from the Danish vHL cohort comprising 165 individuals identified as having vHL in Denmark [45]. The genotypes of the diagnosed Danish vHL families with pathogenetic VHL germline mutations include c.278G > A, c.407 T > C, c.433C > T, c.499C > T, c.319C > T, c.353T > C, c.194C > T, c.337C > T, c.520_521ldupAA, c.293A > G, c.269A > T, c.239G > T, c.194C > G, c.481C > T, c.496G > T, c.463 + 1G > T, c.606dupA, c.194C > G, c.548C > A, c.257C > T, c.500G > A, c.191G > C, c.388G > A and others.

At present, the patient has undergone bilateral adrenalectomy and lifelong hormone replacement therapy. The neuroendocrine tumors of lung and pancreas were confirmed by pathological biopsy. There is no targeted drug for VHL mutation, but the downstream molecular VEGF and other targeted drugs can be selected. At the same time, systemic chemotherapy, immunotherapy and radiotherapy can also affect lung and pancreatic tumors. Moreover, routine genetic and healthy consultation should be carried out for other family members. In case of tumor, registration and genetic monitoring should be carried out, for better constructing the family genealogical map of atypical VHL syndrome and understanding the correlation between genotype and phenotype brought by VHL mutation.

Combined with gene detection and clinical diagnosis, we found that the inherent relationship between VHL genotype and phenotype, and constructed standard diagnosis and treatment process of the disease with rare VHL mutation from the perspective of gene therapy. Future directions mainly focus on exploration of genotype–phenotype relationship, individual therapy and genetic counseling in VHL disease. Because of long-term therapy and specimen storage, we have not performed the relative researches at mRNA and protein levels, therefore exhibiting the limitation of studies on molecular biological mechanisms. Further large population studies, whether clinical or basic, are needed to define the significance of VHL mutation on disease progression, explore potential biochemical and molecular mechanisms, and design large-scale clinical trials.

## Supplementary Information

**Additional file 1**: Somatic mutation RER1 (c.209C>T, p.Ala70Val).

**Additional file 2**: Somatic mutation HMGCL (c.647A>G, p.Asp216Gly).

**Additional file 3**: Somatic mutation KSR2 (c.2720G>A, p.Arg907His).

**Additional file 4**: Somatic mutation PARP4 (c.3509C>T, p.Thr1170Ile).

**Additional file 5**: Somatic mutation VHL (c.500G>A, p.Arg167Gln).

**Additional file 6**: Somatic mutation SCN10A (c.5454G>C, p.Leu1818Phe).

**Additional file 7**: Somatic mutation MSX2 (c.387G>T, p.Met129Ile).

**Additional file 8**: Somatic mutation KIFC1 (c.935G>A, p.Arg312His).

**Additional file 9**: Somatic mutation KPNA7(c.659C>T, p.Thr220Met).

**Additional file 10**: Somatic mutation COL4A6 (c.128C>T, p.Pro43Leu).

## Data Availability

The datasets used and/or analyzed during the current study are available from the corresponding author on reasonable request.
