# Supplementary figures and images for: Identification of a VHL gene mutation in atypical Von Hippel-Lindau syndrome: genotype–phenotype correlation and gene therapy perspective
Source: Cancer Cell Int. 2021 Dec 19;21:685. doi: 10.1186/s12935-021-02386-w (PMC8684656; doi:10.1186/s12935-021-02386-w)

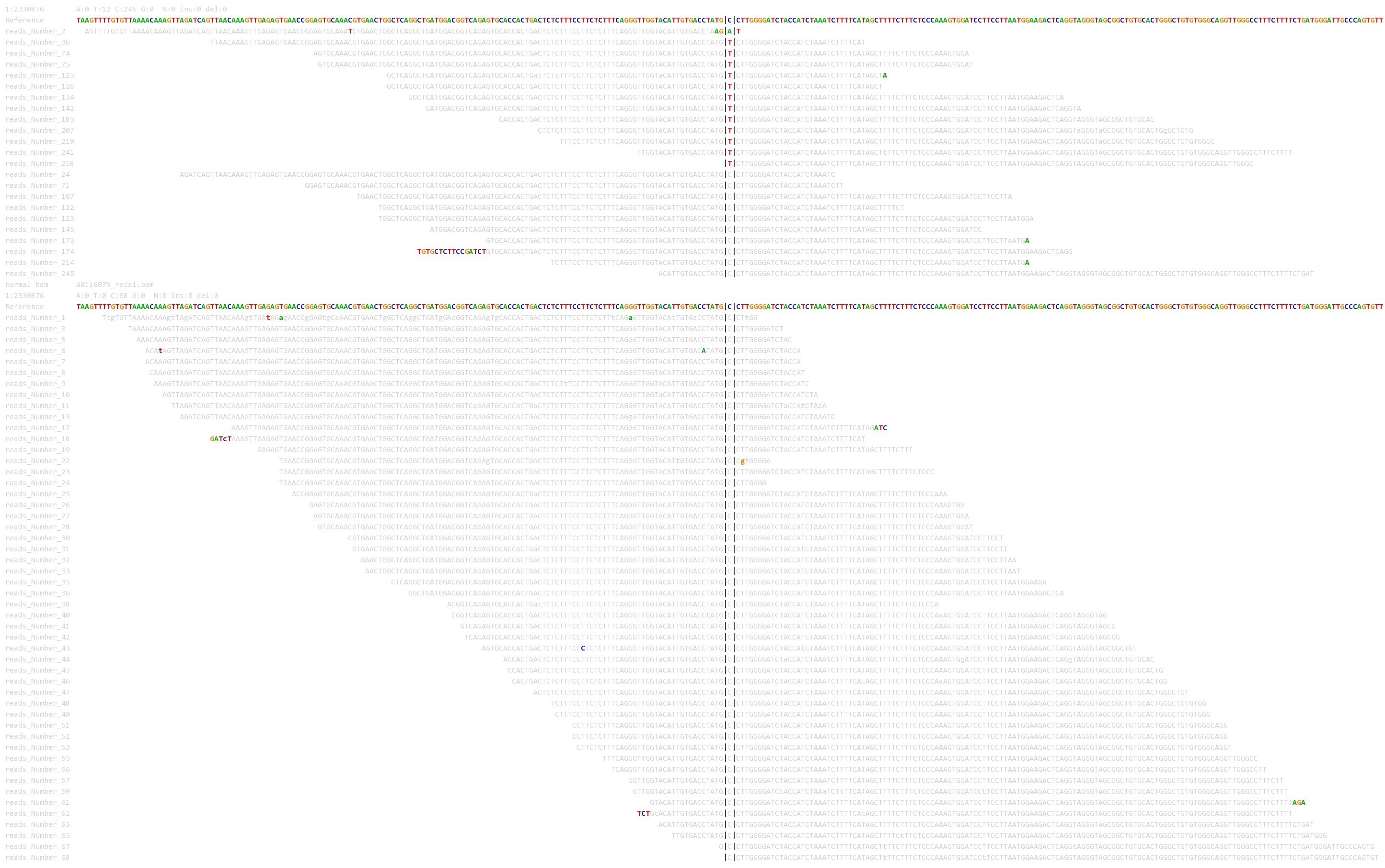

Supplement: Supplementary file 1 — Additional file 1: Somatic mutation RER1 (c.209C>T, p.Ala70Val). [file 12935_2021_2386_MOESM1_ESM.jpg]

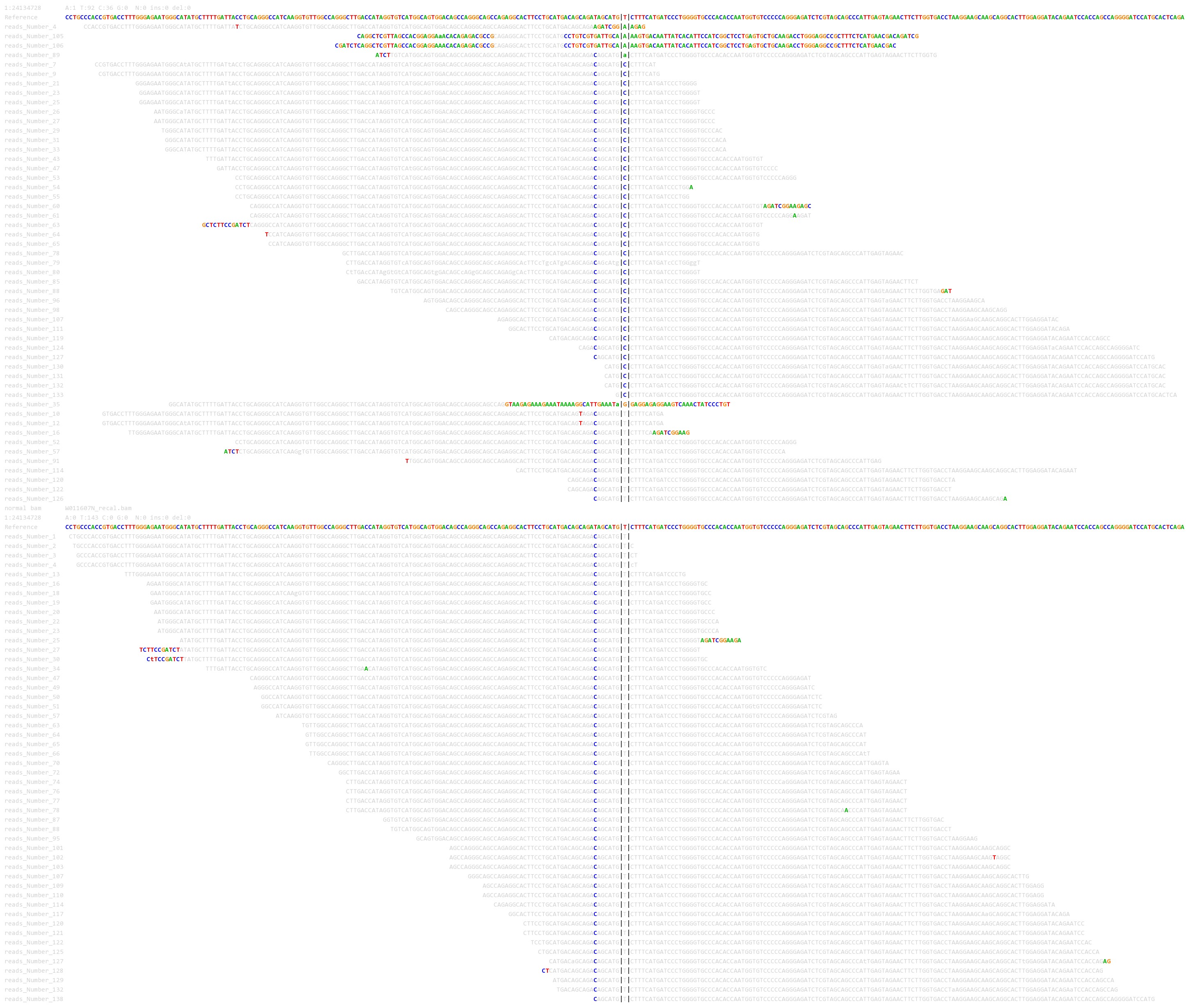

Supplement: Supplementary file 2 — Additional file 2: Somatic mutation HMGCL (c.647A>G, p.Asp216Gly). [file 12935_2021_2386_MOESM2_ESM.jpg]

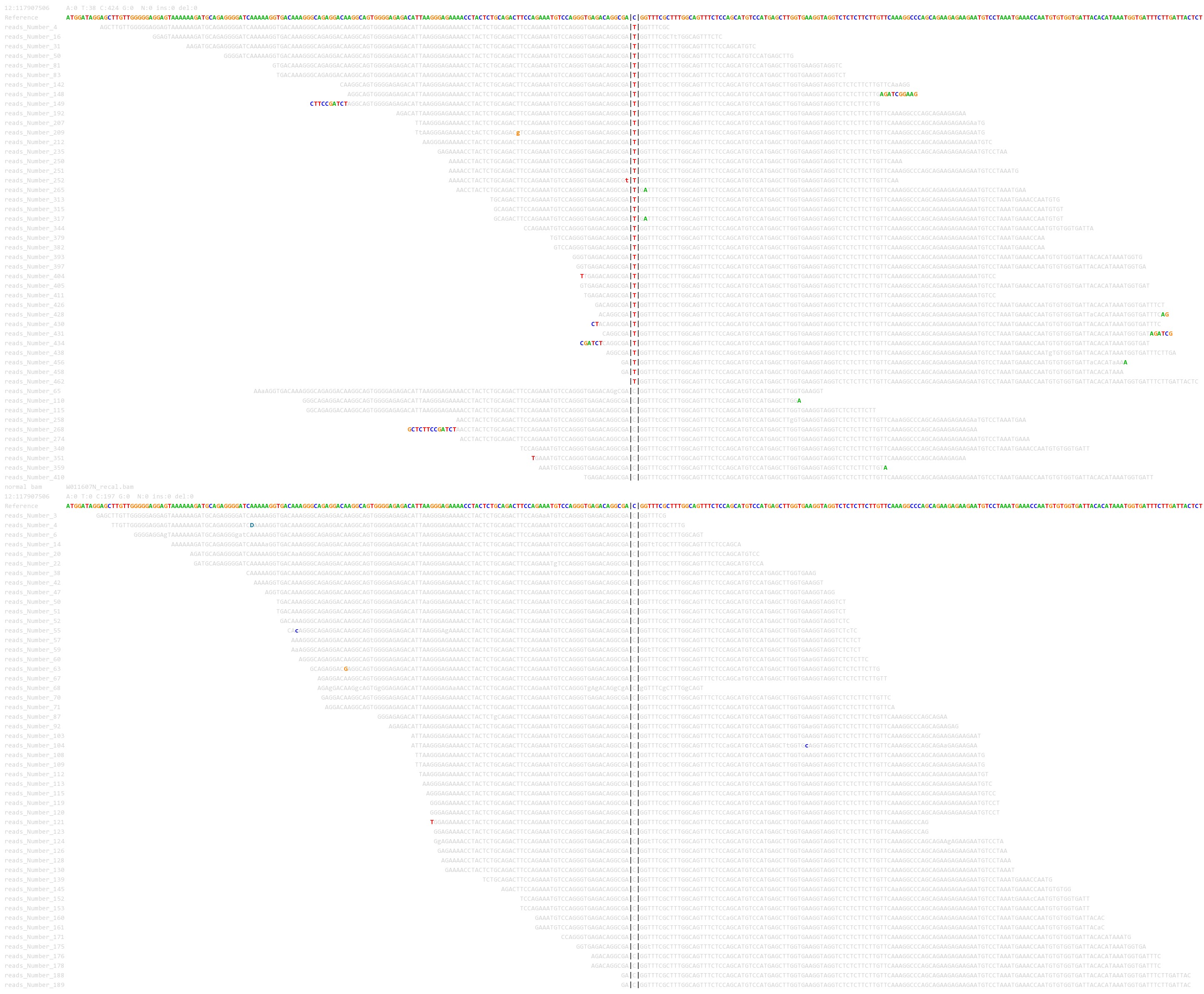

Supplement: Supplementary file 3 — Additional file 3: Somatic mutation KSR2 (c.2720G>A, p.Arg907His). [file 12935_2021_2386_MOESM3_ESM.jpg]

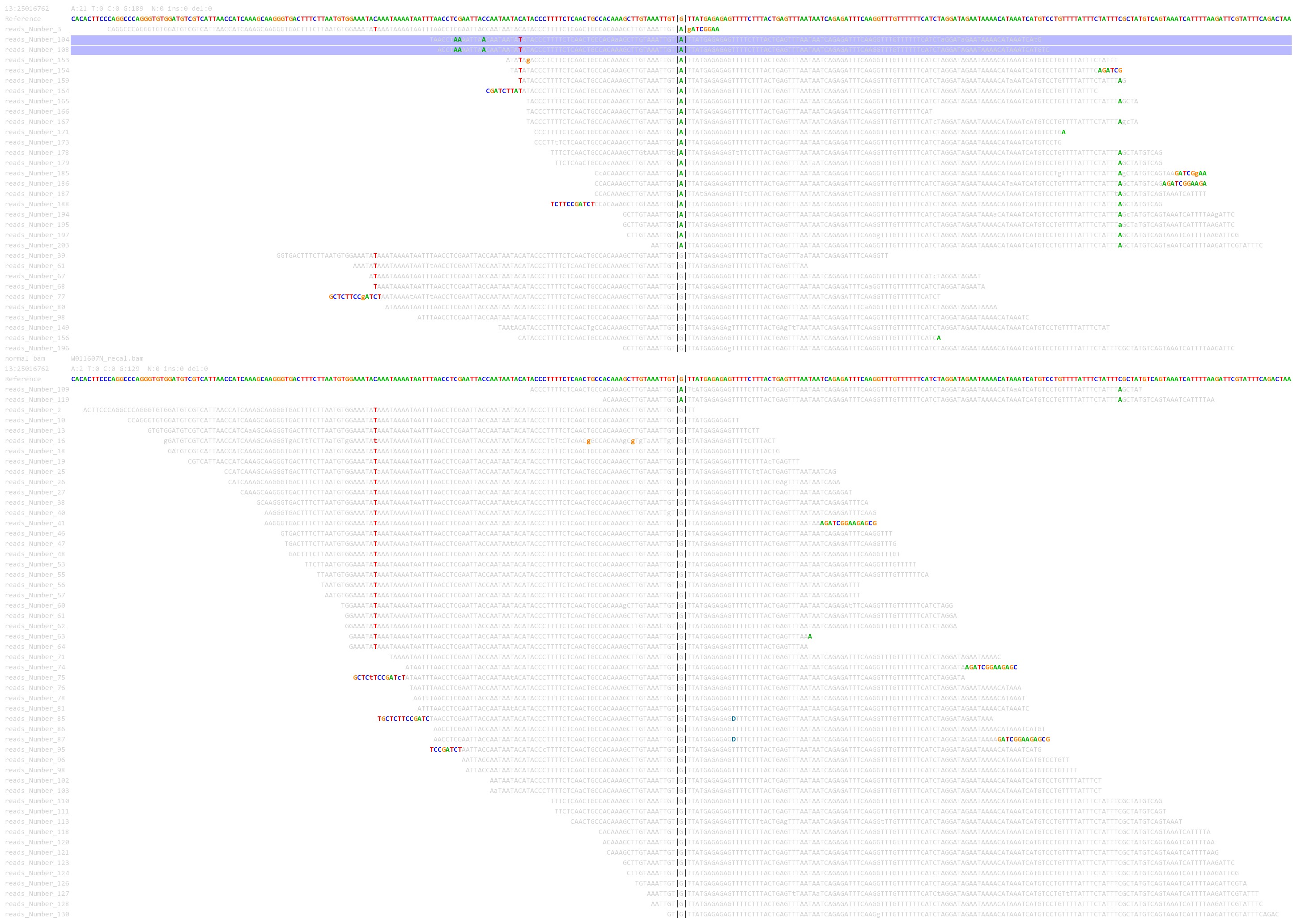

Supplement: Supplementary file 4 — Additional file 4: Somatic mutation PARP4 (c.3509C>T, p.Thr1170Ile). [file 12935_2021_2386_MOESM4_ESM.jpg]

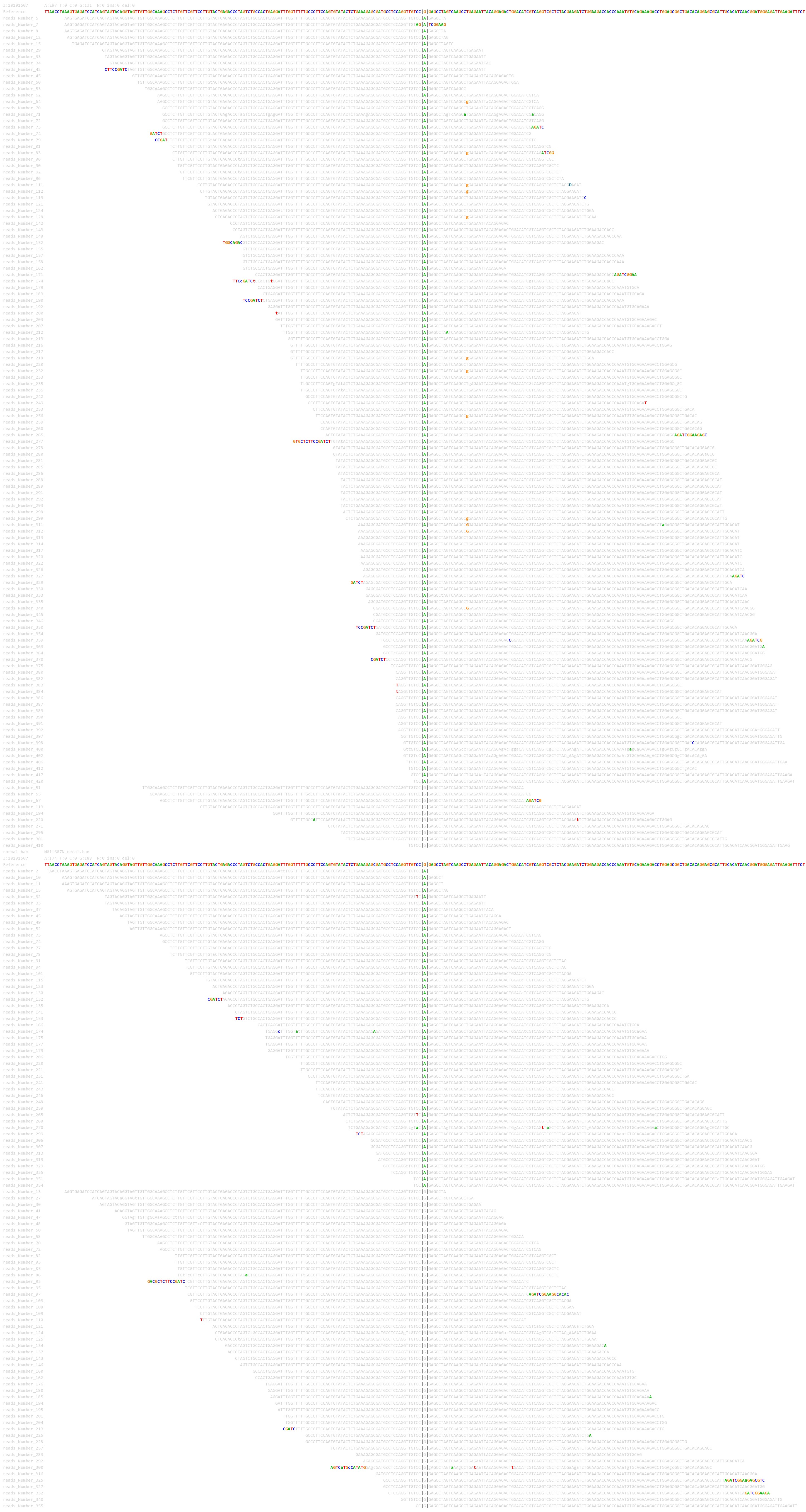

Supplement: Supplementary file 5 — Additional file 5: Somatic mutation VHL (c.500G>A, p.Arg167Gln). [file 12935_2021_2386_MOESM5_ESM.jpg]

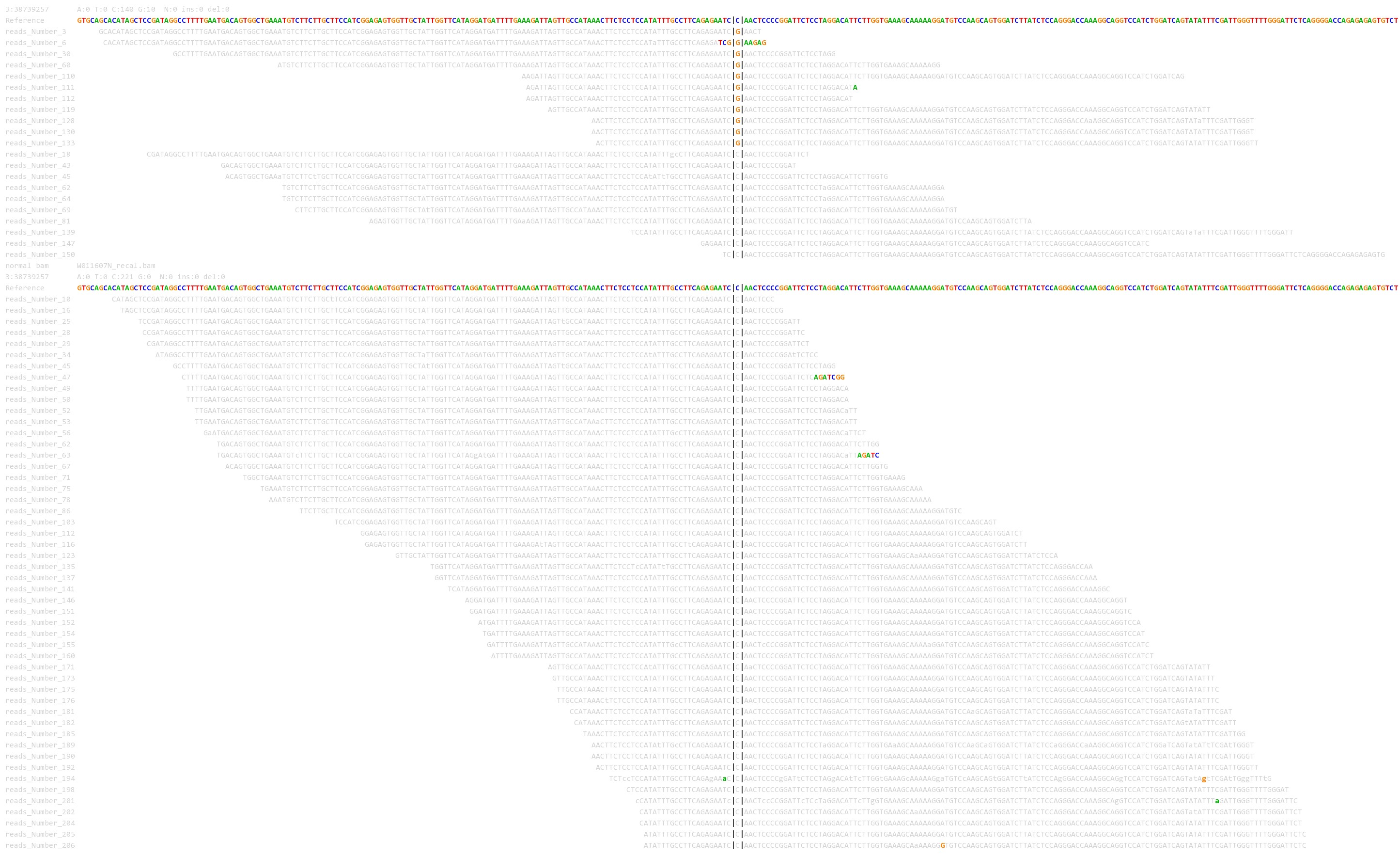

Supplement: Supplementary file 6 — Additional file 6: Somatic mutation SCN10A (c.5454G>C, p.Leu1818Phe). [file 12935_2021_2386_MOESM6_ESM.jpg]

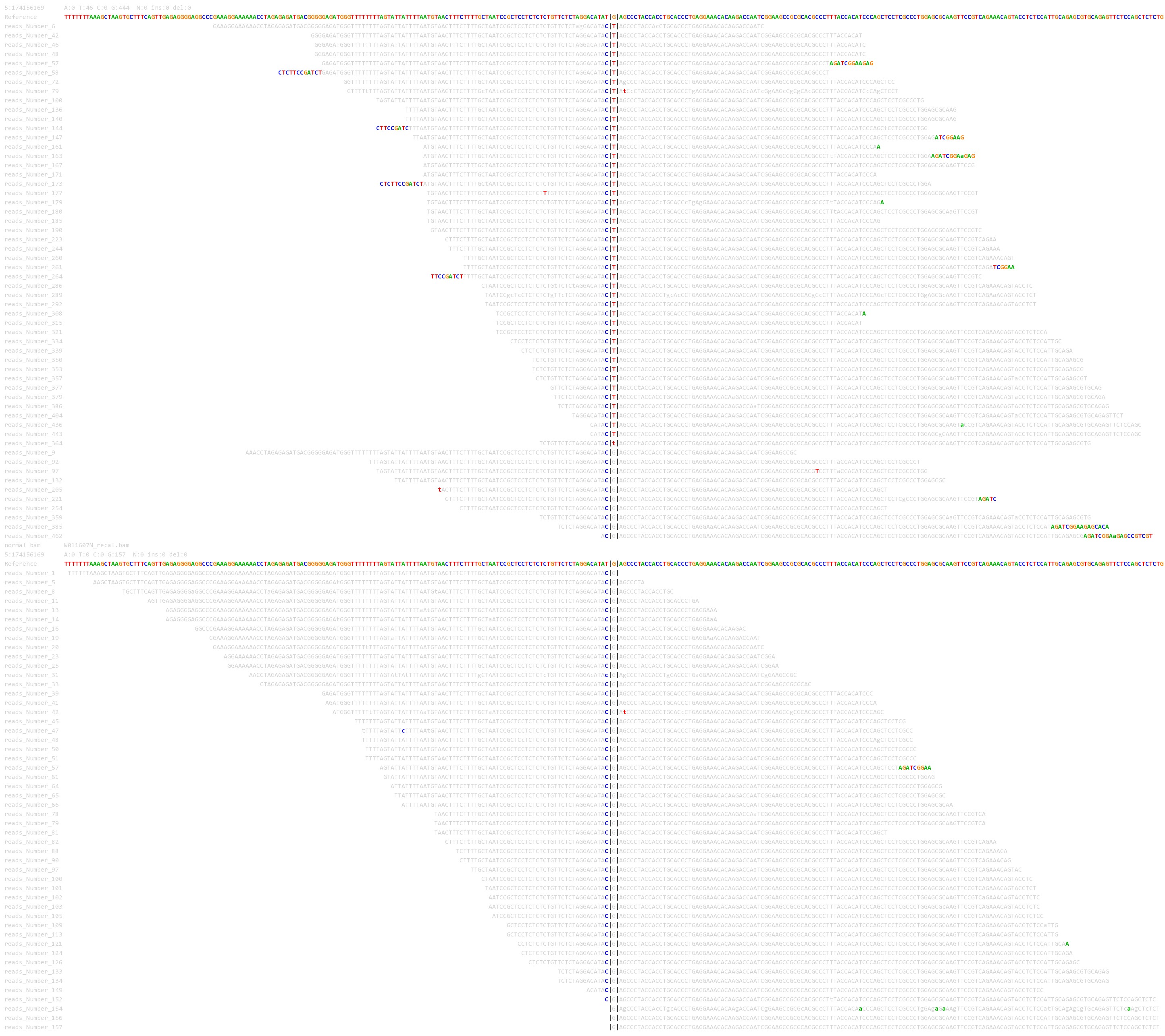

Supplement: Supplementary file 7 — Additional file 7: Somatic mutation MSX2 (c.387G>T, p.Met129Ile). [file 12935_2021_2386_MOESM7_ESM.jpg]

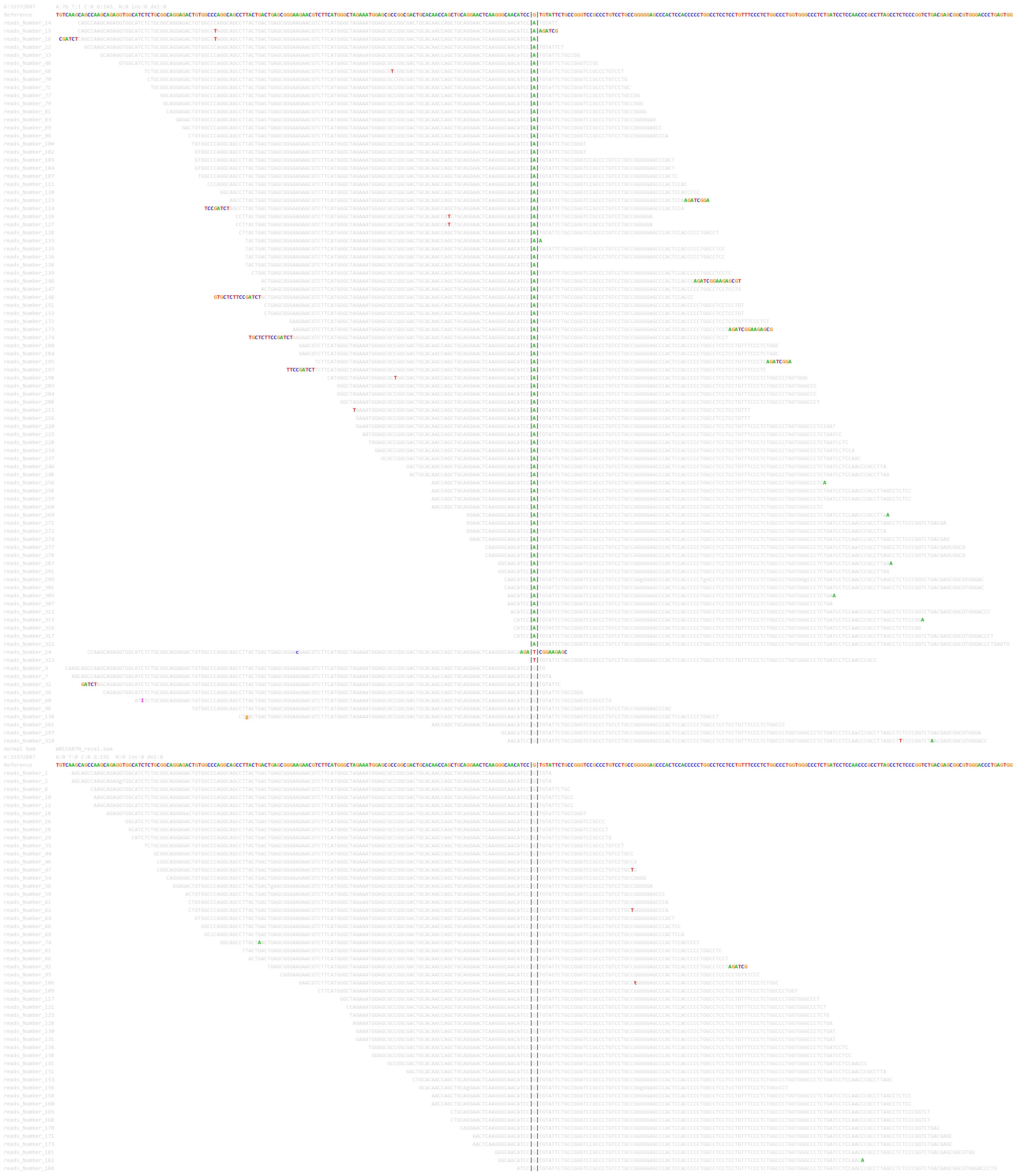

Supplement: Supplementary file 8 — Additional file 8: Somatic mutation KIFC1 (c.935G>A, p.Arg312His). [file 12935_2021_2386_MOESM8_ESM.jpg]

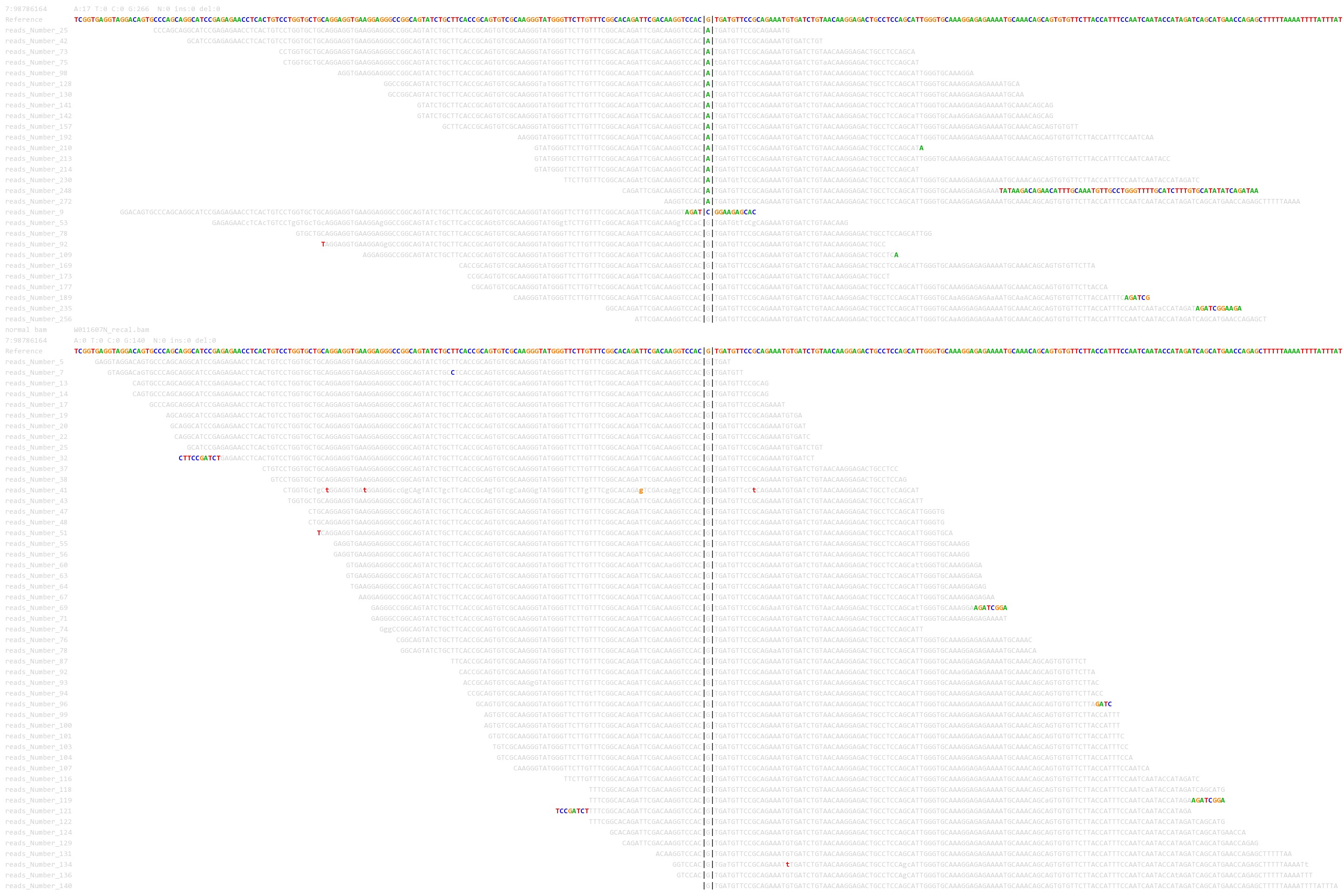

Supplement: Supplementary file 9 — Additional file 9: Somatic mutation KPNA7(c.659C>T, p.Thr220Met). [file 12935_2021_2386_MOESM9_ESM.jpg]

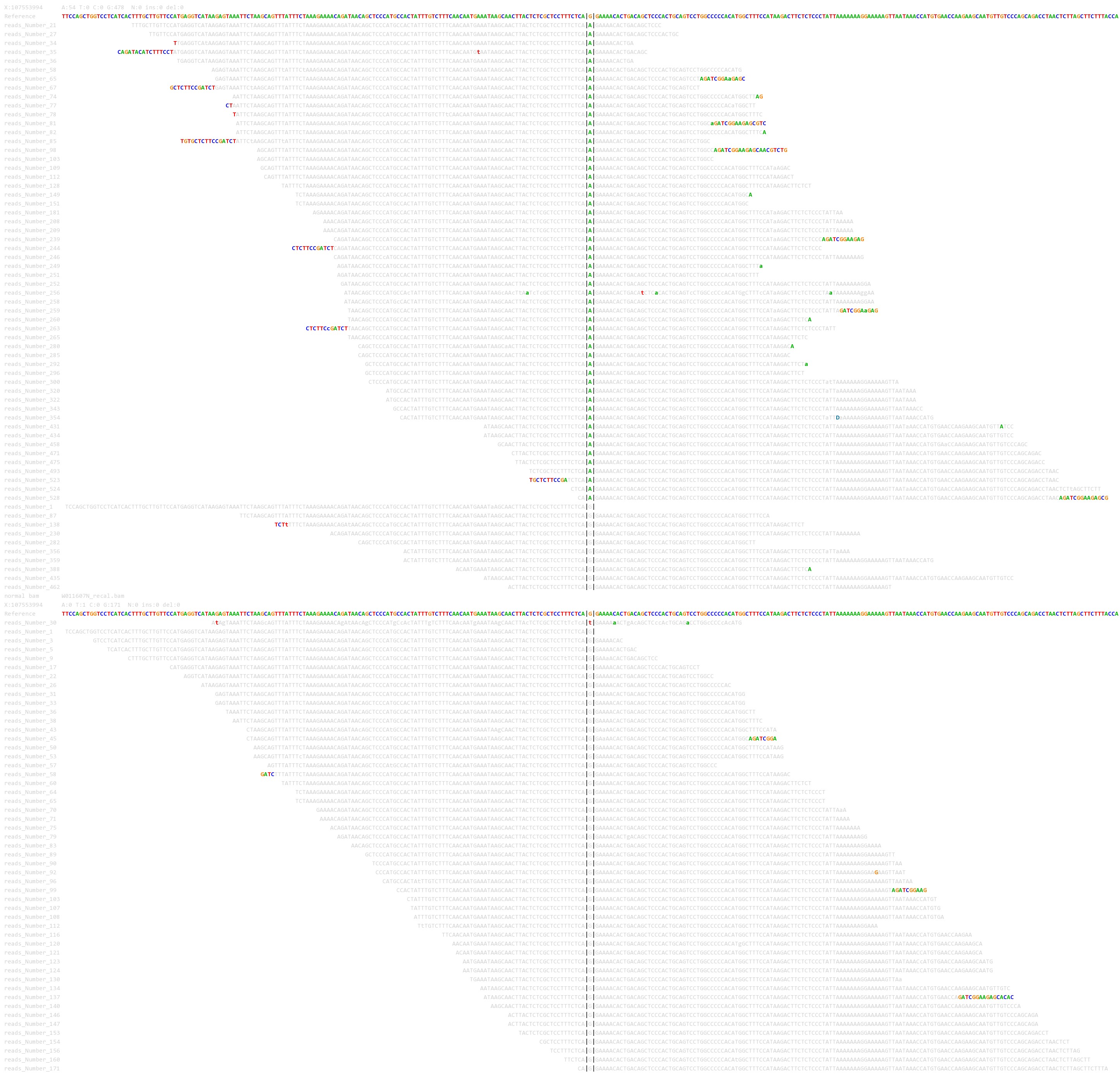

Supplement: Supplementary file 10 — Additional file 10: Somatic mutation COL4A6 (c.128C>T, p.Pro43Leu). [file 12935_2021_2386_MOESM10_ESM.jpg]
